# *Megaselia steptoeae* (Diptera: Phoridae): specialists on smashed snails

**DOI:** 10.3897/BDJ.8.e50943

**Published:** 2020-03-26

**Authors:** Brian V. Brown, Jann E. Vendetti

**Affiliations:** 1 Urban Nature Research Center, Natural History Museum of Los Angeles County, Los Angeles, United States of America Urban Nature Research Center, Natural History Museum of Los Angeles County Los Angeles United States of America

**Keywords:** Natura history, flies, southern California

## Abstract

**Background:**

Phorid flies are amongst the most biologically diverse and species-rich groups of insects. Ways of life range from parasitism, herbivory, fungivory, to scavenging. Although the lifestyles of most species are unknown, many are parasitoids, especially of social insects. Some species of ant-parasitoids are attracted to injured hosts for feeding purposes to develop eggs, as well as for oviposition, requiring each female to find two injured hosts.

**New information:**

Females of the phorid fly *Megaselia steptoeae* Hartop et al. (Diptera: Phoridae) were found to be quickly attracted to crushed glass snails of the species *Oxychilus draparnaudi* (Beck) (Gastropoda: Oxychilidae). Most females were without mature eggs and apparently were attracted for feeding purposes only; other injured molluscs offered at the same time were not attractive. One female laid eggs in captivity and offspring were reared to the pupal stage. The lifestyle of this species is similar to that of parasitoids of injured ants, which also require separate hosts of the same species for feeding and oviposition. We conclude that injured hosts must be common in the environment to attract these host-specific scavengers.

## Introduction

Phorid flies (Diptera: Phoridae) are a group of small (0.4-4.0 mm long) insects that are species-rich and common worldwide. The species diversity of phorid flies from urban Los Angeles, California, is becoming well-known thanks to the Biodiversity Science: City and Nature (BioSCAN) project (Brown and Hartop 2016, Hartop et al. 2015, Hartop et al. 2016a, Hartop et al. 2016b), but the life histories of most phorid species are still unknown. This lack of information inhibits the interpretation of measures of abundance, diversity and species composition (e.g.Adams et al. in press, McGlynn et al. 2019). As phorids are amongst the most biologically diverse families of insects, they cannot be categorised simply as “scavengers” (or some other convenient generalisation), as many have extremely specific parasitoid or other lifestyles (Disney 1994, Brown 2018). Unfortunately, it is not a simple matter to encounter new life histories (Hartop et al. 2018), with serendipity playing an important role (as in Brown and Hartop 2017).

**Figure 1.**
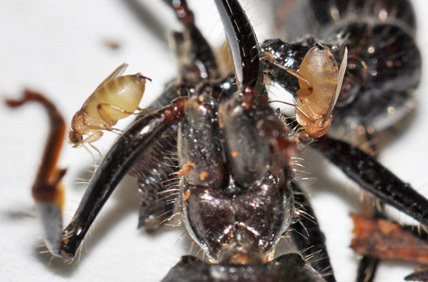
Bloated, non-gravid “feeder” females of *Apocephalus paraponerae* Borgmeier on an injured Paraponera clavata F. ant at La Selva Biological Station, Costa Rica (photo by Brian Brown).

Many phorid flies are parasitoids of injured hosts (Brown and Feener DH Jr. 1991a, Brown and Feener DH Jr. 1991b, Brown and Feener DH Jr. 1993, Brown 2000, Brown 1997, Feener DH Jr. et al. 1996, Hash 2014, Brown et al. 2015). These hosts must be injured before being acceptable, presumably because healthy hosts are too active or too dangerous. Generally, only females are attracted, although they arrive at hosts *in copula* with males, which then depart (males were not encounterd in the observations documented in this paper, however). Nearly all of these female flies also feed on their hosts, often imbibing haemolymph until they appear bloated (Fig. 1); some are able to release excess fluid through anal secretions during feeding. Many of these flies have no mature eggs in their bodies and could not possibly be present for oviposition purposes (dubbed “feeders” by Brown 2000), but a smaller fraction immediately begins to lay eggs (‘layers’). This behaviour is common in species that exist in apparently large populations that arrive promptly when hosts are injured, especially *Apocephalus paraponerae* Borgmeier, a parasitoid of the giant tropical ant *Paraponera clavata* F. in South and Central America (Brown and Feener DH Jr. 1991a, Brown and Feener DH Jr. 1991b). Boren (2016) found that *A. paraponerae* females attracted to hosts were 70% feeders and 30% layers, whereas another host-feeding species, *Apodicrania molinai* Borgmeier were 93% layers and 7% feeders. In the ant-parasitoids, flies are attracted to hosts through olfactory cues, especially alarm pheromones (Feener DH Jr. et al. 1996, Morehead and Feener 2000), with possibly the same type of chemicals attractive to parasitoids of injured millipedes (Hash 2014, Hash et al. 2017) and possibly bees, although such studies have not yet been done. Hosts are attractive to parasitoids for a brief period of time, genrerally about one-half of an hour, presumably the time during which they produce the attractive pheromones. Afterwards, when the host dies, the parasitoids are no longer attracted.

Herein, we report on another record of a relatively unrelated phorid fly attracted to injured hosts, with intriguing similarities to the lifestyle of ant, millipede and bee parasitoids.

## Materials and methods

Flies were attracted by crushing live adult snails and placing them on dead leaves on the ground. Specimens were collected into 95% alcohol and one specimen was sequenced by Sanger sequencing. Museum specimens of flies were slide-mounted in Canada balsam after clearing in clove oil. One snail was sequenced (GenBank Accession # MN734267), using the methods of Vendetti et al. (2018). Voucher flies are deposited in the collection of the Natural History Museum of Los Angeles County and a CO1 barcode sequence of one specimen is in the BOLD database as POTW001-19.

## Taxon treatments

### Megaselia steptoeae

Hartop, Brown, & Disney, 2015

ED6E9A00-8A20-5799-AFBB-2074ADAD64B0

#### Ecology

**Figure 2.**
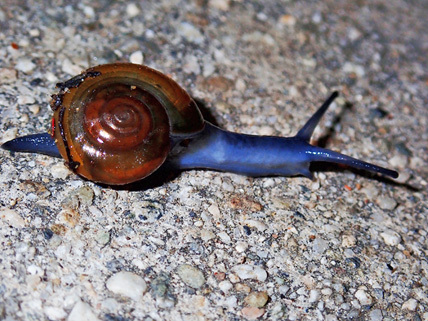
Living individual of *Oxychilus draparnaudi* (Beck).

**Figure 3.**
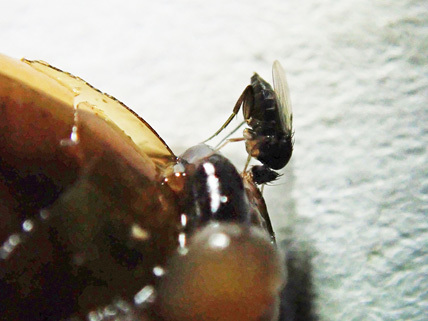
Female *Megaselia steptoeae* Hartop et al. feeding on crushed *Oxychilus draparnaudi* (Beck).

While trying to attract another species of the extremely species-rich genus *Megaselia* Rondani, at the Los Angeles County Arboretum, we made an unexpected discovery. Females of *M. steptoeae* were attracted to crushed individuals of the glass snail *Oxychilus draparnaudi* (Beck); Mollusca: Oxychilidae) (Fig. 2). These tiny (1.5 mm long) flies were collected at the Arboretum on two occasions and, in each case, they arrived at the injured glass snail quickly after we crushed the snails (40 s and 13 s, respectively). (Fig. 3). On 15 August 2018, we collected five flies and on 9 August 2019, we collected 14 flies. As all flies were females and morphological identification of *Megaselia* species usually requires male specimens, the five flies from 2018 were sequenced to provide identifications. Identification of the female specimen was made by comparing CO1 sequences (DNA barcodes) of known male specimens of *M. steptoeae* (Biodiversity Index Number BOLD:AAP4678) with those of the snail-attracted females. Of the specimens collected in 2019, three were kept alive in rearing containers with crushed snails, one of which laid eggs near the snails (the actual oviposition was not observed). Two individual larvae appeared to be mature after four days and were reared to the pupal stage, after which they died. On both occasions in the field, we also provided baits of European garden snails (*Cornu aspersum* (Müller); Mollusca: Helicidae) and various slugs, none of which attracted any phorid flies. Like the ant hosts, injured glass snails were attractive to flies for a relatively brief period of time (about one-half hour).

A third collecting event took place at the Natural History Museum of Los Angeles County Nature Garden on 17 August 2019. Three snails were crushed, attracting a single female *M. steptoeae* in ten minutes. One of the snails was barcoded to verify the identification as *O. draparnaudi*.

## Discussion

*Oxychilus draparnaudi* is a predatory land snail, native to Western and South-western Europe that has been introduced worldwide, including to California, likely through the horticulture trade (Robinson 1999, Roth and Sadeghian 2006). It is most often found in human-inhabited and anthropogenically-altered environments, wherein it can become quite common (Robinson 1999).

*Megaselia steptoeae* was one of 30 new species of phorid flies described from urban Los Angeles (Hartop et al. 2015). They are small (1.5 mm body length), dark brown flies that are widespread in the Los Angeles area, being found in 28 of the 30 sites surveyed (Brown and Hartop 2016). Besides Los Angeles, there are specimens of *M. steptoeae* in the BOLD database from southern Florida, USA, Argentina, Belize, Costa Rica and Mexico (http://v3.boldsystems.org/index.php/Public_BarcodeCluster?clusteruri=BOLD:AAP4678).

In North America, *Oxychilus draparnaudi* is considered to be an invasive species, originally from the Palearctic Region. Based on records in BOLD and other large-scale Malaise trapping studies, *M. steptoeae* has not been found there, at least not in Germany (Geiger et al. 2016, Morinière et al. 2016), which might be outside of the main range of the species (SW and W Europe, according to Welter-Schultes 2012) . If the interaction between these two species is one that only occurred after transport of the snails to North America, it is only about 100 years old and is a product of human commerce.

It is unknown if *Oxychilus draparnaudi* releases alarm pheromones, but such compounds have been identified in sea slugs as methyl ketones (Sleeper and Fenical 1977) and polypropionates (Marin et al. 1999). Various alarm responses due to the presence of crushed conspecifics have been observed in marine (Atema and Stenzler 1977, Jacobsen and Stabell 2004), freshwater (Ichinose et al. 2003) and terrestrial gastropod taxa (Pakarinen 1992). These types of behaviour likely indicate the release of chemical cues at injury or death in diverse snail species.

There are few studies on development in female phorid flies, so we are almost completely ignorant of whether flies are autogenous (able to mature eggs based on resources gained through larval feeding) or anautogenous (requiring adult feeding to mature a batch of eggs). The little available information is summarised by Disney (1994). In most cases, it is impossible to determine from the records whether feeding on protein-rich haemolymph was required to allow eggs to develop and mature. Some species require mating before eggs mature, whereas others do not. In the wild, female parasitoid phorids are not normally known to feed away from hosts, with exceptions being observations in Gregor (1977) , who recorded 2 females of *Apocephalus ecitonis* Borgmeier on carrion and two females of *Myriophora juli* (Brues) from human faeces. In our experience, however, with hundreds of injured ants and other arthropods exposed in the field, only parasitoids of the injured host species are attracted to feed.

One of the hypotheses for the origin of parasitism is that scavenging species, attracted to dead or dying hosts for feeding purposes, become more and more aggressive, eventually attacking the hosts before they are injured (Eggleton and Belshaw 1993). Our observations are not, however, consistent with this hypothesis. Obligate feeding restricted to their oviposition hosts suggests that these flies evolved host choice (and parasitism) before host feeding; otherwise, it would be in the fly’s best interest to feed at any available source of arthropod haemolymph, in the case of the ant, bee and millipede parasitoids or any crushed molluscs, in the case of the snail feeders. This interpretation is further supported by phylogenetic information on *Apocephalus* Coquillett, the largest group of ant parasitoids in the family, in which species with host feeding and the utilisation of injured hosts, arise well within the genus and are not in the earliest diverging lineage (Brown et al. 2018). We find it striking that such similar parallel behaviour occurs in a relatively distantly-related metopinine genus as *Megaselia*, in which we assume it has evolved separately.

Species with this type of life history demonstrate the continuum of lifestyles embraced by the terms “scavenger” and “parasitoid”. The definition of a scavenger is an organism that consumes dead or decaying organic material (Lincoln et al. 1998), whereas a parasitoid is one that feeds on a single host, ultimately causing its death (as opposed to predators that feed on more than one host or true parasites that feed on a host, but do not kill it - Godfray 1994). The situation with these flies is intermediate, as they attack animals that are going to die (and do not cause their death), yet are not attracted to the hosts past a short window of acceptability.

A requirement for two injured hosts, one for feeding, one for egg-laying, would seem to make the parasitoid (or specialised scavenger) lifestyle even more risky than it already is, doubling the need for females to find hosts. If such hosts are extremely common in the environment, however, such a strategy is sustainable. Indeed, the existence of species that require two hosts is a strong argument that such injured hosts are abundant in the environment.

## Supplementary Material

XML Treatment for Megaselia steptoeae
